# Experiencing social exclusion changes gut microbiota composition

**DOI:** 10.1038/s41398-022-02023-8

**Published:** 2022-06-17

**Authors:** Chong-Su Kim, Go-Eun Shin, Yunju Cheong, Ji‑Hee Shin, Dong-Mi Shin, Woo Young Chun

**Affiliations:** 1 Department of Food and Nutrition, College of Human Ecology, Seoul National University, Seoul, 08826 Republic of Korea; 2 Department of Psychology, Chungnam National University, Daejeon, 34134 Republic of Korea; 3 Research Institute of Human Ecology, Seoul National University, Seoul, 08826 Republic of Korea

**Keywords:** Psychology, Physiology

## Abstract

Gut microbiota is suggested to regulate the host’s mental health via the gut-brain axis. In this study, we investigated the relationship between the microbiome and psychological pain due to social exclusion. Adult individuals with (*n* = 14) and without (*n* = 25) social exclusion experience were assessed for the psychological status using self-reported questionnaires: Beck Anxiety Inventory (BAI), Beck Depression Inventory, and the UCLA Loneliness Scale. The gut microbiota was analyzed by 16 S rRNA gene sequencing and bioinformatics. The exclusion group had a 1.70-fold higher total BAI score and 2.16-fold higher levels of anxiety-related physical symptoms (*p* < 0.05). The gut microbial profiles also differed between the two groups. The exclusion group showed higher probability of having *Prevotella*-enriched microbiome (odds ratio, 2.29; 95% confidence interval, 1.65–2.75; *p* < 0.05), a significantly reduced Firmicutes/Bacteroidetes ratio, and decreased abundance of *Faecalibacterium* spp. (*p* < 0.05) which was associated with the duration and intensity of social exclusion (*p* < 0.05). Our results indicate that the psychological pain due to social exclusion is correlated with the gut microbiota composition, suggesting that targeting social exclusion-related microorganisms can be a new approach to solving psychological problems and related social issues.

## Introduction

Psychological pain defined as an internal response to noxious psychological stimuli [1] is mainly caused by social exclusion or rejection [2], leading to other mental problems [3]. Indeed, several studies reported that individuals with an experience of social exclusion or rejection are vulnerable to social conflicts and psychiatric diseases, including bipolar disorder and schizophrenia [4, 5]. Moreover, it has been suggested that there is a correlation between psychological pain and suicidality [1, 6]. For example, a study on potentially suicidal patients indicates that individuals with increased intensity of psychological pain may be at a higher risk for a suicide attempt [7]. However, despite the critical role of the psychological pain in various mental health problems, studies on the objective criteria to assess the psychological pain and its effects on human health are limited.

People who never experienced social exclusion tend to underestimate the severity of psychological pain [8]. However, it has been suggested that the psychological pain can be as strong as the physical pain and may even increase the intensity of the latter [1, 9]. Furthermore, emerging evidence suggests that the psychological pain can be transcribed into the physical pain [9, 10], because there is a substantial overlap between the neuroarchitectures involved in the feeling of social pain and the sensation of physical pain [1, 9]. For example, polymorphisms in the µ-opioid receptor-encoding gene, which is strongly associated with physical pain, have been reported to be involved in the experience of social pain due to experimentally induced social rejection [11]. Furthermore, a neuroimaging study that used functional MRI to examine human brain reactions to the psychological pain caused by social rejection indicates that the cerebral regions processing physical pain are activated during emotional pain [12]. Overall, these findings provide compelling evidence of the link between the psychological and physical pain, suggesting that social pain needs to be assessed and considered for both psychological and physical health aspects. However, this area of research still remains at a relatively early stage of development.

It is established that the gut microbiota, a community of microorganisms found in the gastrointestinal tract, has pivotal roles in various aspects of host physiology [13, 14]. Recent discoveries suggest that gut microbes can affects the functional activities of the host’s brain, including emotions, cognition, and behavior through the gut-brain axis [15–20]. Thus, changes in the gut microbiome structure have been continuously reported in people with psychological disorders, suggesting the role of intestinal microorganisms in human mood and behavior. Several studies have reported that patients suffering from depression have abnormal gut microbial composition compared to healthy individuals [21, 22]. Germ-free mice devoid of all microorganisms show decreased immobility time in the forced swimming test, whereas transplantation of fecal microbiota from patients with major depressive disorder results in depression-like symptoms, indicating that shifts in the microbial community can cause anxiety- as well as depression-related behavior [21]. It is also reported that depression and dysphoria in people with borderline personality disorder are linked to alteration in the gut microbiome associated with increased oxidative stress and inflammatory responses, and changes in the opioidergic activity [23]. More recently, mounting evidences support the fact that microbial reconstitution by fecal microbiota transplantation and probiotic treatment alleviate the social deficits in rodent models of autism spectrum disorders [24, 25]. However, despite the current evidence of the link between gut microorganisms and social behavior, there are no population studies that assessed the association between the psychological pain and the intestinal microbial community.

Therefore, the aim of the present study was to test a hypothesis that changes in the gut microbiota could be related to the psychological status in individuals who experienced social exclusion.

## Materials and methods

### Study design and participants

This case-control study was conducted at Chungnam National University (Daejeon, Republic of Korea) from June 2016 to June 2017. All procedures followed the ethical standards of the 1964 Helsinki declaration and its later amendments. The study was approved and monitored by the Institutional Review Board of Chungnam National University (IRB No. 201604-BR-017-01) and written informed consent was obtained from all participants. This work is registered with CRiS (Clinical Research Information Service; http://cris.nih.go.kr; Registration ID: KCT0006920).

The participants were recruited from communities of the Daejeon city through recruitment flyers posted at Chungnam National University. Onsite candidate screening was performed by interviews, during which the information about health history, health-related behavior, and social exclusion experience was collected. Eligible participants were young adults (18–32 years) who provided consent to be assigned to one of the study groups. Assessment of the social exclusion experience and current psychological status was performed by trained research staff. Individuals who used any dietary supplements (including pre/probiotics), antibiotics, and gastrointestinal medications within the past 3 months were excluded, as these factors could affect the quality of the gut microbiome data. People with specific dietary habits such as vegetarian diet, and frequent alcohol and caffeine uses were also excluded. After screening for eligibility, a total of 39 participants were enrolled in the study and assigned to either the control or exclusion group. The participants visited the clinic once for psychological assessment, and fecal samples were collected at that time.

### Assessment of the psychological status

The psychological pain and social exclusion status were assessed using several approaches. First, the number, duration, and intensity of social exclusion episodes were recorded through single-item self-report questionnaires containing the following questions: “If you have had an experience of social exclusion: ‘How many times of social exclusion experience have you had?’; ‘How long have you experienced social exclusion?’; and ‘How was the intensity of the social exclusion experience?’” The three answers should have been frequency, duration (months or years), and the score from 1 (not at all) to 10 (very severe), respectively.

Anxiety was assessed using the Korean version of Beck Anxiety Inventory (BAI) consisting of 21 self-reporting multiple-choice questions for rating the intensity of physical, emotional, and cognitive symptoms such as numbness, tingling, and fear of the worst observed during the past week [26, 27]. Each item was scored on the scale from 0 (not at all) to 3 (severe) and the total score was used to determine the severity of anxiety.

Depression was evaluated based on the Korean version of Beck Depression Inventory (BDI) [28, 29] consisting of 21 self-reporting questions related to depression symptoms such as hopelessness, feelings of being punished, and fatigue experienced during the past week [30]. The symptoms were scored on the scale from 0 (not at all) to 3 (severe) and the total score was used to evaluate the severity of depression.

The level of loneliness was assessed using the Korean version of UCLA Loneliness Scale, a 20-item questionnaire aimed to determine how often the person felt disconnected from the others [31, 32]. Each item was scored on the scale from 1 (never) to 4 (always).

### Gut microbiota analysis

#### Fecal sample collection

Each participant was provided with a stool collection kit, which included collection tube, flushable collection sheet, nitrile gloves, and a cooler bag, and asked to follow the manufacturer’s instructions. Fecal samples were supposed to be collected within a 24 h period before the visit, stored at 4 °C, then placed in a cooler bag and brought at the time of the visit to Chungnam National University. The samples were immediately placed at −20 °C and then shipped in a bag filled with dry ice to Seoul National University (Seoul, Republic of Korea), where sample aliquots (180~200 mg) were immediately stored at −80 °C until analysis of the gut microbiome.

#### Genomic DNA extraction

Total bacterial DNA was isolated from stool samples by using the QIAamp® fast DNA Stool Mini Kit (QIAGEN, Hilden, Germany) according to the manufacturer’s instructions, and examined for quality and quantity by gel electrophoresis and spectrophotometry (NanoDrop ND-2000, Thermo Scientific, Waltham, MA, USA), respectively.

#### Amplification and sequencing of 16 S rRNA genes

Hypervariable regions (V3–V4) of the 16 S rRNA gene were amplified by PCR in a total volume of 50 μl containing 0.25 μl of BioFact F-Star Taq DNA polymerase (BioFACT™, Seoul, Republic of Korea), 20 ng of DNA template, 5 μl of 10× Taq buffer (20 mM Mg^2+^), 1 μl of 10 mM dNTP mix, and 2 μl of forward and reverse barcoded primers (10 pmol/μl). PCR was performed in a GeneAmp® PCR system 9700 (Applied Biosystems, Foster City, CA, USA) under the following thermocycling conditions: initial denaturation at 94 °C for 5 min, followed by 28 cycles of denaturation (30 s, 95 °C), annealing (30 s, 60 °C), and extension (30 s, 72 °C), a final extension step at 72 °C for 10 min, and cooling to 4 °C. PCR products were confirmed by electrophoresis in 1% agarose gels, visualized using a Gel Doc system (BioRad, Hercules, CA, USA), purified with PureLink Quick Gel Extraction and PCR Purification Combo Kit (Invitrogen, Carlsbad, CA, USA), and quantified using a Qubit 2.0 fluorometer (Invitrogen). The size of the libraries was assessed using BioAnalyzer (Agilent Technologies, Santa Clara, CA, USA). The amplicons of participants were sequenced in an Illumina MiSeq sequencing system (Illumina, San Diego, CA, USA).

#### Bioinformatic analysis of sequencing data

Bioinformatic analysis was conducted as previously describe [33]. Microbial sequences were processed using QIIME1 (version 1.9.0) [34]. Briefly, sequences were denoised to remove chimeras and samples with low quality scores using DADA2, clustered into Operational Taxonomic Units (OTUs), and representative OTU sequences were aligned according to the SILVA database (version 132) at the 99% sequence identity with scikit-learn Naïve Bayes-based machine-learning classifier. A phylogenetic tree was generated using MAFFT and FastTree method for diversity analyses. Downstream analysis of alpha diversity was carried out to assess differences in richness and evenness of microbial communities. Comparison of the relative abundance of various microorganism was performed to reveal differences in microbial profiles across the samples.

### Dietary intake analysis

Dietary intake was evaluated using a 3-day food record method, a standardized tool for dietary assessment. Participants were asked to record the types, amounts, ingredients, and cooking methods of the food they consumed in the previous 3 days (including 2 weekdays and 1 weekend day). Total energy, carbohydrate, protein, and fat intakes were analyzed by using the Computer Aided Nutritional Analysis Program (CAN-Pro 5.0, Korean Nutrition Society).

### Statistical analysis

The sample size was determined to ensure adequate power for statistical significance using the G-Power 3.1 with α level of 0.05 and desired power of 0.08. All statistical analyses were conducted using Partek (version 6.6; Partek, Saint Louis, MI, USA) and SPSS (version 25.0; SPSS Inc., Chicago, IL, USA). The normality assumption and homogeneity of variance were tested by Kolmogorov-Smirnov test for study variables. To compare variables between the groups, we performed independent *t*-test or Fisher’s exact tests. Correlations were assessed by Spearman’s rank correlation analysis. Correction for multiple testing was performed based on the False Discovery Rate (FDR). The *p*-value < 0.05 and FDR < 0.05 were considered to indicate statistical significance in all tests.

## Results

### Demographic and psychological characteristics of the participants

A total of 39 participants enrolled in the study were assessed for the experience of social exclusion during their lifetime. Based on the results from self-reports, those who never experienced interpersonal problems were assigned to the control group (*n* = 25), whereas those who had experienced social exclusion were assigned to the exclusion group (*n* = 14). Demographic and psychological characteristics of the participants are summarized in Table 1. There was no difference between the control and exclusion group in the average age of the participants (21.48 and 21.79 years, respectively; *p* = 0.466) or the male to female ratio (*p* = 0.266). The participants in the exclusion group had in average 1.54 social exclusion episodes of moderate intensity (average score 5.07), which lasted about 11.59 months (Table 1).

**Table 1:** Demographic and psychological characteristics of participants.

General characteristics	Control (*n* = 25)	Exclusion (*n* = 14)	*P*-value
**Male/Female (*N*)**	9/16	2/12	0.266
**Age (yrs)**	21.48 (2.28)	21.79 (3.62)	0.466
**Social exclusion experience**			
**Number of exclusions (N)**	.	1.54 (0.66)	<0.001
**Duration of exclusion experience (months)**	.	11.59 (10.96)	0.002
**Intensity of exclusion experience (scores)**	.	5.07 (2.17)	<0.001

Data are presented as mean (SD). *P*-values based on Fisher’s exact test or t-test.

### Psychological pain due to social exclusion

To verify the effects of social exclusion on the psychological pain, we further assessed the psychological status of our participants based on the signs of anxiety, depression, and feeling of loneliness. Anxiety was evaluated using BAI, which covers psychological symptoms such as feeing unsteady, scared, and nervous (Cronbach’s α = 0.870) and physical symptoms such as numbness or tingling, dizziness, indigestion, and hot/cold sweats (Cronbach’s α = 0.882). As shown in Table 2, the severity of the anxiety-related psychological symptoms tended to be higher in the exclusion group than in the control group (8.71 ± 6.37 *vs*. 6.32 ± 5.39; *p* = 0.220), whereas that of the anxiety-related physical symptoms was significantly (2.16 times) higher (9.21 ± 6.83 *vs*. 4.26 ± 4.07; *p* = 0.024). Interestingly, among the physical signs, the severity of bowel symptoms such as indigestion and numbness was significantly increased in the exclusion group compared to the control group (data not shown). The total BAI score was 1.70 times higher in the exclusion group than in the control group (17.93 ± 11.61 *vs*. 10.54 ± 8.11; *p* = 0.022). Furthermore, the symptoms of depression and loneliness measured by BDI and UCLA Loneliness Scale were more severe in the exclusion group, although the difference with the control group was not statistically significant (all *p* > 0.05, Table 2). Collectively, these results indicated that social exclusion increased the psychological pain and intensified physical symptoms related to anxiety.

**Table 2:** Psychological pain status of participants.

Psychological pain index	Control (*n* = 25)	Exclusion (*n* = 14)	*P*-value
**Beck Anxiety Inventory (scores)**	10.17 (8.08)	17.93 (11.61)	0.022
**Psychological symptoms**	6.32 (5.39)	8.71 (6.37)	0.220
**Physical symptoms**	4.26 (4.07)	9.21 (6.83)	0.024
**Beck Depression Inventory (scores)**	11.88 (10.53)	16.62 (10.63)	0.198
**UCLA Loneliness Scale (scores)**	40.96 (11.23)	46.21 (9.48)	0.148

Data are presented as mean (SD). *P*-values based on t-test.

### Comparison of the gut microbiota profiles

**Fig. 1:**
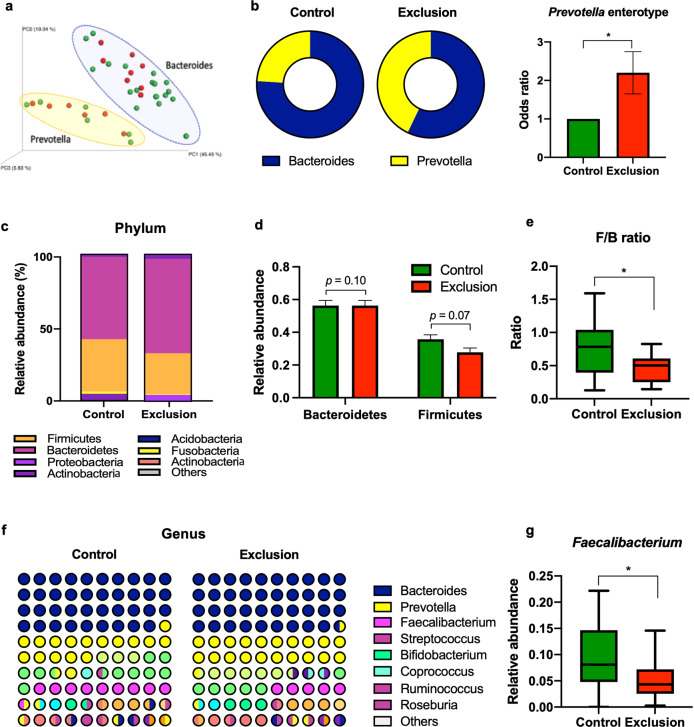
Gut microbiota profiling in individuals with and without social exclusion experience. **a** Principal Coordinate Analysis (PCA) plot of microbiome samples from the control and exclusion groups. **b** Pie charts showing the percentage of *Bacteroides* or *Prevotella* spp. in the samples from the control and exclusion groups. The bar graph shows odds ratios and the 95% confidence interval for the probability of having *Prevotella*-enriched enterotype in the exclusion group compared to the control group. **c** Relative abundance (%) of the phyla in the two groups of participants shown as a stacked bar graph. **d** Relative abundance of the two most abundant phyla Bacteroidetes and Firmicutes shown as a bar graph. **e** The ratio of Firmicutes to Bacteroidetes shown as a box-and-whisker plot. **f** Relative abundance of bacteria at the genus level in the control and exclusion groups shown as a dot plot matrix. **g** Relative abundance of the genus *Faecalibacterium* shown as a box-and-whisker plot. The lowest and highest points in a box-and-whisker plot correspond to the minimum and maximum of the data set, respectively, and the horizontal line in the middle indicates the mean value. *P < 0.05 according to *t*-test.

To determine whether the gut microbiota composition was associated with social exclusion experience, we analyzed fecal samples from each individual for the bacterial community structure by 16 S rRNA pyrosequencing. Principal coordinate analysis (PCA) carried out based on the microbiota composition identified two distinct microbiome clusters in the whole study population, which were dominated by the genera *Bacteroides* and *Prevotella*, respectively (Fig. 1a). Interestingly, we found that compared to the samples of the control group, those of the exclusion group were enriched with *Prevotella* (24.00% *vs*. 42.86%), which resulted in a higher probability of having *Prevotella*-enriched microbiome for individuals suffering from social exclusion (odds ratio [OR] 2.29; 95%, confidence interval [CI] 1.65–2.75, *p* < 0.05; Fig. 1b). The finding that social exclusion cases were not equally distributed between the two microbial clusters prompted us to examine the abundance of intestinal bacteria at the phylum and genus levels in the two groups of participants. The results indicated that there were slight between-group differences in two most representative phyla: Bacteroidetes tended to be more abundant, whereas Firmicutes – less abundant in the exclusion group compared to the control group (Fig. 1c, d), although the difference was not statistically significant. However, the Firmicutes/Bacteroidetes ratio was significantly reduced in the exclusion group compared to the control group (0.46 ± 0.21 *vs*. 0.74 ± 0.42; *p* < 0.05, Fig. 1e). As according to previous findings, the Firmicutes/Bacteroidetes ratio is often related to dietary factors, we analyzed the 3-day dietary records and daily nutrient intake in the control and exclusion groups to reveal possible effects of the diet on the microbial community structure; however, there were no differences in the total energy and major nutrients intake between the groups (Supplementary Table 1). Collectively, these results indicated that the Firmicutes/Bacteroidetes ratio might be affected by the psychological status independently of dietary factors.

Analysis of the gut microbial changes at the genus level showed that the exclusion group had a significantly reduced abundance of bacteria belonging to the genus *Faecalibacterium* (0.05 ± 0.39 *vs*. 0.09 ± 0.06; *p* < 0.05, Fig. 1f, g).

### Correlation between Faecalibacterium and psychological pain from social exclusion

**Fig. 2:**
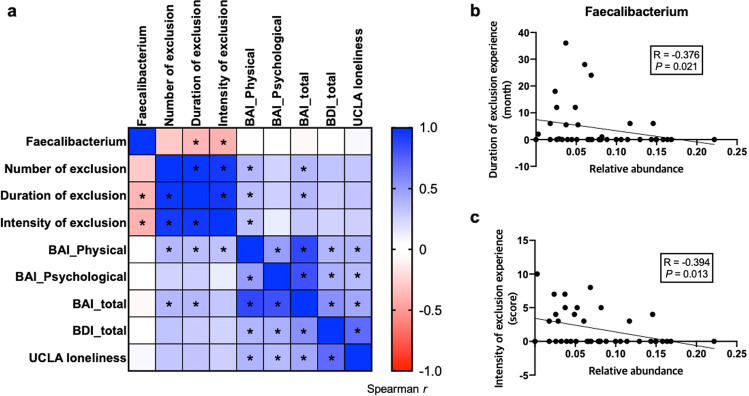
Association between Faecalibacterium and psychological pain caused by social exclusion. **a** Heatmap of the correlation matrix between the relative abundance of *Faecalibacterium* and psychological indexes. Colors in the heatmap indicate Spearman’s correlation coefficient and asterisks indicate statistical significance (*p* < 0.05). **b**, **c** Significant negative correlation of *Faecalibacterium* relative abundance with the duration (**b**) and intensity (**c**) of the exclusion experience; Spearman’s correlation coefficient (R) and the level of statistical significance (*p*) are indicated. Each dot represents the results for an individual participant.

Next, we assessed the correlation between the relative abundance of *Faecalibacterium* showing a significant between-group difference and all indexes of psychological pain due to social exclusion (Fig. 2a). The results revealed that the relative abundance of *Faecalibacterium* spp., which was reduced in the exclusion group, was negatively correlated with the duration of the social rejection period (*R* = −0.376, *p* = 0.021; Fig. 2b) and the intensity of exclusion experience (*R* = −0.394, *p* = 0.013; Fig. 2c). These results suggest that the genus *Faecalibacterium* comprises a group of intestinal bacteria most affected by the psychological pain due to social exclusion.

We also found that the increased level of anxiety-related physical symptoms in the exclusion group was significantly positively associated with the number, duration, and intensity of social exclusion experience, and the BDI and UCLA loneliness scores (Fig. 2a), supporting our hypothesis that the psychological pain and physical pain caused by social exclusion are closely interconnected.

## Discussion

The relationship between the functional activity of the brain and the gut microbiota has received great attention during the past decades, resulting in the elucidation of the role of intestinal microorganisms in the host’s mood and behavior. However, there are still gaps in our understanding of the relationship between the microbiome and psychological status because of certain experimental challenges; one of the most important is the difficulty to identify and recruit individuals with psychological issues such as social pain and then to analyze their mental status in parallel with microbial composition. Therefore, the majority of studies at the brink of psychology and microbiology have been performed on animal models such as rodents, which have limitations in terms of extrapolating the results to humans. In this cross-sectional case-control study, we could perform analysis of the relationship between the psychological state and gut microbiome of individuals with social exclusion experience, which is the strength of the current investigation.

Anxiety is a normal adaptive response of mind and body to threats, which prepares the organism to an emergency situation [35]. However, if anxiety persists for a long time, it has negative effects not only on the mental state but also on physical health. Thus, several studies have reported that long-term anxiety results in the deterioration of cardiovascular, immune, and gastrointestinal functions [36–38]. In line with these findings, we revealed that individuals who experienced social exclusion had stronger anxiety-related physical symptoms, confirming the direct link between the psychological and physical pain. Furthermore, the symptoms of depression and loneliness (BDI and UCLA scores) tended to be more severe in the exclusion group, although the difference with control was not statistically significant. Since people generally underestimate the severity of psychological pain, much more attention needs to be paid to the impact of social exclusion on mental health, including anxiety and depression.

The correlation between physical and mental health is further supported by our finding that the microbiomes of the control group were enriched with *Bacteroides* spp., whereas those of the exclusion group – with *Prevotella* spp. This result is consistent with the notion that the enterotype, which is classification of the intestinal microbiome, is closely associated with the emotional status [39, 40]. Thus, a recent study has shown that gut microbial composition in individuals with the *Prevotella*-dominant enterotype was significantly associated with positive emotions, whereas no such relationship was found in those with the *Bacteroides* enterotype [40]. It has also been reported that healthy adults in the *Prevotella*-dominant group have stronger response to emotional stimuli in the limbic system, a brain region involved in the emotional process [39]. In line with these findings, the present study suggests that the gut microbiome plays a key role in shaping the psychological profile of the host.

Furthermore, we found that individuals who suffered from social rejection had a reduced Firmicutes/Bacteroidetes ratio compared to the control group. Shifts in the Firmicutes/Bacteroidetes ratio have long been recognized as a relevant marker of gut dysbiosis implicated in several health issues such as obesity and anxiety- and depression-like behaviors [41–43]. A decrease in the Firmicutes/Bacteroidetes ratio has been detected in mice susceptible to social defeat stress, in which it was associated with stress-induced depression-like behavior; at the same time, normalization of the Firmicutes/Bacteroidetes ratio has resulted in antidepressant-like effects through effects on the immune system [44]. These findings, along with our data, suggest an association between the Firmicutes/Bacteroidetes ratio and the psychological status of individuals suffering from social pain.

The present study showed that the exclusion group had reduced abundance of bacteria belonging to the *Faecalibacterium* genus, which was significantly associated with the psychological pain due to social exclusion. These findings are consistent with the correlation between *Faecalibacterium* and anxiety and depression disorders revealed in earlier studies. For example, the abundance of *Faecalibacterium* spp. has been found to be positively associated with quality of life score [45] and negatively associated with symptoms of depression: patients with depressive disorder had reduced *Faecalibacterium* levels compared to healthy individuals [22]. It has been shown that *Faecalibacterium* spp. are butyrate-producing gut bacteria which can suppress intestinal inflammation [46] and affect mood and behavior via the gut-brain axis [47]. In an animal study, the administration of *Faecalibacterium prausnitzii* has significant anxiolytic and antidepressant effects by modulating cytokine production: increasing the levels of anti-inflammatory interleukin (IL)-10 and decreasing those of pro-inflammatory IL-6, suggesting a critical role of *Faecalibacterium* in psychiatric disorders [48]. Moreover, recent studies have reported that reduction in the gut-derived butyrate level has negative impacts on mitochondrial melatonin synthesis which thereby affects various psychiatric conditions including depression [49, 50]. Butyrate also takes part in epigenetic regulation of the genes related to cognition and anxiety via inhibition of histone deacetylases [51]. Therefore, our findings indicate that alterations in the butyrate-producing *Faecalibacterium* abundance may dysregulate the gene transcription related to the brain functions, suggesting that changes in *Faecalibacterium* could serve as biomarkers of psychological pain caused by social rejection.

Furthermore, the changes in the microbiome of the exclusion group observed in this study may be indicative of a general shift toward increased inflammation, which is supported by the finding that bowel symptoms such as indigestion were significantly more severe in the exclusion group. Increasing evidence points on the role of inflammation in the correlation between psychological pain and gastrointestinal disorders. It is believed that psychological factors are directly implicated in the stimulation of pro-inflammatory processes which ultimately lead to functional problems in the gastrointestinal tract [19, 52]. For example, it has been reported that the prevalence of psychiatric disorders in patients with irritable bowel syndrome is associated with the severity of bowel symptoms such as diarrhea and alterations in the gut microbiome, especially increased abundance of *Blautia* spp. [53]. There is also a link between abnormal intestinal permeability caused by shifts in gut microbiota and psychiatric disorders. According to the leaky gut hypothesis, the intestinal barrier function is deregulated through changes in the gut microbial composition, leading to increased intestinal permeability and subsequent infiltration of pathogenic bacteria and their metabolites into systemic circulation where they are detected by the host immune system; as a result, the secretion of pro-inflammatory cytokines is increased and inflammation activated [54]. A recent cross-sectional study has documented changes in gut permeability markers zonulin and intestinal fatty acid-binding protein in patients with a recent suicide attempt, which show correlation with the levels of pro-inflammatory IL-6 [55]. Another study has demonstrated that an increase in intestinal permeability (measured by the lactulose/mannitol ratio) is associated with the severity of depression in unmedicated adolescents [56]. Collectively, these findings showing the link between gut microorganisms, inflammation, and psychological disorders are consistent with our results, suggesting that anxiety-related intestinal symptoms could be attributed to the compositional changes in the intestinal microbiome and activation of systemic inflammation. However, future studies are needed to establish the causative relationship and underlying mechanisms.

The present study had some limitations. Although the statistical significance of the results was confirmed, the probability level was marginal, which could be attributed to the small sample size and decreased statistical power. It is quite challenging to recruit people exposed to social exclusion situations, as indicated by previous clinical studies on social exclusion that were performed on samples of similar or smaller sizes [57, 58]. Therefore, future studies using larger samples are warranted to validate our findings. In addition, this was a cross-sectional study in which it is not possible to determine the causal link between psychological markers and gut microbiota. Further investigations should provide the mechanistic insights into the crosstalk between the gut microbiota and the psychological status.

Despite these limitations, the present study shows that the psychological pain caused by social exclusion is associated with the alterations of the microbial community in the gut. This work provides evidence of physiological interconnection between gut microbiota and mental health, suggesting that therapies targeting the social exclusion-related microbiota can be used as a new approach to relieve mental sufferings and solve related social issues.

## Supplementary information

Supplementary table 1

## Data Availability

The data presented in this study are available upon request from the corresponding author.
