# Supplementary material for: Experiencing social exclusion changes gut microbiota composition
Source: Transl Psychiatry. 2022 Jun 17;12:254. doi: 10.1038/s41398-022-02023-8 (PMC9205890; doi:10.1038/s41398-022-02023-8)
Supplement: Supplementary file 1 — Supplementary table 1 [file 41398_2022_2023_MOESM1_ESM.docx]

**Supplemental Material**

**Supplemental Table 1. Daily dietary intake**

| **Daily dietary intake** | **Control (n = 25)** | **Exclusion (n = 14)** | **P-value** |
| --- | --- | --- | --- |
| Total energy (kcal) | 1861.64 (698.84) | 1713.28 (595.36) | 0.53 |
| Carbohydrate (g) | 250.30 (84.09) | 252.46 (81.43) | 0.94 |
| Total protein (g) | 65.36 (33.41) | 59.91 (21.80) | 0.54 |
| Animal protein (g) | 39.19 (28.23) | 31.50 (16.09) | 0.38 |
| Vegetable protein (g) | 26.17 (9.77) | 31.53 (10.61) | 0.14 |
| Total fat (g) | 63.04 (34.98) | 57.43 (18.16) | 0.60 |
| Animal fat (g) | 32.30 (23.45) | 24.65 (17.34) | 0.32 |
| Vegetable fat (g) | 30.60 (15.79) | 37.37 (17.81) | 0.26 |
| Dietary fiber (g) | 16.50 (10.35) | 12.70 (5.39) | 0.23 |

Data are presented as mean (SD). P-value based on t-test.
